# The Murmurs of Aortic Incompetence

**Published:** 1907-05-04

**Authors:** Cecil Wall

**Affiliations:** Assistant Physician to the London Hospital and to the Brompton Hospital for Diseases of the Chest


### HOSPITAL CLINICS.

### THE MURMURS OF AORTIC INCOMPETENCE.

By CECIL WALL, M.A., M.D.Oxon., M.R.C.P.Lond., Assistant Physician to the London Hospital and to the Brompton Hospital for Diseases of the Chest.

Lecture delivered at the Hospital for Consumption and Diseases of the Chest, Brompton.

### PRODUCTION OF A MURMUR.

I HAVE selected for discussion to-day a point which is of considerable clinical interest and concerned with many of the problems arising in connection with the auscultation of the diseased heart.

In a closed system of tubes containing moving fluid, such as the arterial system, vibrations having some regularity and periodicity may be set up when the calibre of the containing channel at any point is abruptly increased. The moving liquid under these conditions is said to form an "internal fluid vein." If the flow is sufficiently swift the vibrations may be transmitted to the surrounding solids and may become audible, and the sound produced, being to a certain extent regular and periodic, partakes of the nature of music rather than of noise, and is termed "a murmur."

### PRODUCTION OF A MURMUR IN AORTIC INCOMPETENCE.

If for any reason the aortic valves cease to be competent the physical conditions necessary for the production of an audible murmur are likely to be fulfilled. During the diastolic period of each cycle blood flows back from the comparatively narrow aorta into the comparatively wide ventricle, and under the combined influences of the elastic recoil of the stretched arterial system, the effect of gravity, and the aspirating power of the ventricle, the flow usually is sufficiently swift to produce vibrations which are transmitted to the ear as sonorous waves. Thus we explain the production of a murmur when there is diastolic regurgitation of blood through the aortic valves.

### CAUSES OF INCOMPETENCE OF THE AORTIC VALVES.

We may now pass to the consideration of the pathological conditions under which the aortic valves may become incompetent.

1. *Dilatation of Arch.*—The simplest condition, though by no means the commonest, is that in which there is such dilatation of the commencement of the aorta that the coaptation of the semilunar cusps is no longer possible. Aneurysmal dilatation of the ascending part of the arch of the aorta (diffuse or saccular) due to atheroma or to syphilitic arteritis is the most usual cause of such incompetence. It is said that such dilatation may be in some instances acute and transient. The proof of this statement seems to be still lacking.

2. *Malformation of the Valves.*—When the aortic valves are incompetent the cusps are usually at fault.

(i.) *Congenital Malformation.*—Congenital malformation of the aortic valves associated with incompetence is extremely rare; though the cusps may be abnormal, yet generally they are able to perform their function.

(ii.) *Acute Endocarditis.*—Acute inflammatory changes affecting the cusps may lead to incompetence in three ways. (1) Vegetations may form on the cusps and obstruct their closure. This may occur in malignant or progressive endocarditis. In simple endocarditis the vegetations are seldom of sufficient size to produce incompetence. Vegetations leading to regurgitation may at times become detached so that the valve again becomes competent. (2) The inflammatory change may lead to ulceration and to loss of tissue of the cusps. Perforation or complete destruction of one or more of the cusps may be the consequence. This condition is met with most commonly in progressive or malignant endocarditis. (3) Inflammation may be followed by cicatrisation and deformity of the valves. This is the commonest cause of incompetence when the valvulitis is of rheumatic origin. The free margin of the valve is the first part affected, the process spreading hence towards the base. Not uncommonly the border of the cusp is fibrous or even calcareous, while the attached portion is still supple.

(iii.) *Degenerative Changes.*—Atheromatous changes developing in the aorta not infrequently spread to the cusps of the aortic valves, and so lead to deformity and incompetence. Syphilitic arteritis may produce a similar effect. In this case the change is first found at the attached portion of the cusps, the free part often remaining for a long time comparatively supple. The condition is often associated with dilatation of the first part of the arch of the aorta, and not uncommonly the rigid projecting base of the valve leads to some narrowing of the aortic orifice.

(iv.) *Rupture of Valve Segments.*—Possibly a healthy cusp may sometimes be ruptured as a result of a sudden rise of blood-pressure consequent upon great exertion. Most usually, when this accident occurs, it is found that the valve had been previously damaged by disease.

### AUSCULTATORY SIGNS.

Our next consideration must be the auscultatory signs found in examining a heart of which the aortic valves are incompetent.

(*a*) *The Second Sound.*—In the first place I wish to draw your attention for a moment to the second sound of the heart. It has been shown recently that the second sound is not caused by the closure of the semilunar cusps, but by the sudden tension to which they are submitted at the commencement of the ventricular diastole, that is, when the over-distended arterial system drives the blood back forcibly against the valves as the elastic recoil comes into play. If the cusps are so badly damaged by disease that they are no longer capable of being thrown into vibration, the second sound cannot be produced. In many cases of aortic incompetence this occurs, especially in those in which acute inflammation is the ætiological factor. In such cases absence of the second sound generally indicates extensive disease and consequently extensive regurgitation. To prevent confusion with the pulmonary second sound, the auscultation for the aortic second sound should be made over the great vessels in the neck. In other cases, though the valve is undoubtedly incompetent, the second sound is heard, and may be even louder than usual; this indicates that the cusps are not completely destroyed. It occurs most frequently when the regurgitation is due to dilatation of the arch or to a spread of degenerative processes into the bases of the cusps. The persistence of the second sound when the valve is incompetent owing to dilatation of the aorta disposes of the view that the second sound is produced by the snapping together of the cusps, and confirms the view that it is due to the tension caused by the reflux of blood when the systole has ceased.

(*b*) *Time of the Murmur.*—The murmur which generally indicates incompetence of the aortic valve begins as soon as the ventricle enters upon its diastole, and generally lasts throughout that period, If the second sound is audible the murmur may be heard following it.

(*c*) *Propagation of the Murmur.*—If the murmur be loud it will be heard at the junction of the second right costal cartilage with the sternum or in the second intercostal space (the aortic area), over the lower half of the sternum, over that part of the chest which corresponds with the area of superficial cardiac dullness, and even in the axilla. In many cases the area of audibility is not so wide; the murmur may be only audible at the inner end of the third and fourth left intercostal spaces. Sometimes it can be heard only at the lower end of the sternum, sometimes only in the third and fourth right intercostal spaces. If the murmur is difficult to hear it may be made louder by raising one or both arms of the patient. This, by the aid of gravity, increases the rate of the regurgitant flow.

(*d*) *Character of the Murmur.*—The murmur in aortic incompetence is usually soft and blowing in character, gradually dying away before the succeeding systolic first sound. Sometimes it is loud and vibratory, producing a palpable thrill. Occasionally it has a distinctly musical character. No constant pathological conditions have been found to explain these variations.

*The Austin Flint Murmur.*—Occasionally it is found that in cases of aortic incompetence without a mitral lesion the murmur, instead of being soft and fading away during the diastole, has a vibrating character and gets louder until it ends suddenly in the systolic sound. This murmur in short may possess all the characters of the presystolic murmur which we usually associate with obstruction of the mitral orifice. In such cases the presence or absence of mitral stenosis may be extremely difficult to determine. This diastolic murmur having the clinical characters of that commonly heard in mitral obstruction, was originally described by Austin Flint and bears his name. Several explanations have been offered. Flint supposed that the regurgitant blood from the aorta floats up the mitral segments, so that they are close together at the commencement of the auricular systole. This floating of the segments he suggested causes a virtual stenosis of the mitral orifice, and so gives the conditions necessary for the production of a presystolic murmur. Broadbent thinks that the blood regurgitating from the aorta impinges on the anterior cusp of the mitral valve, throws it into vibration, and so produces the murmur. Maguire supposes that the currents of blood from the aorta and the left auricle which flow on either side of the anterior mitral cusp are moving with unequal rapidity, and so throw the cusp into vibration. For myself, I think that none of these explanations seems probable. I have noticed that a murmur, presystolic in time and vibratory in character, is apt to appear in cases of aortic incompetence when the left ventricle shows signs of dilatation, disappearing when compensation is restored. A similar murmur also appears in patients suffering from chronic interstitial nephritis when the left ventricle begins to fail and in some cases of adherent pericardium, when there is no obstruction of the mitral valve. The common factor in these three instances seems to be a dilated ventricle with a comparatively strong auricle. I venture to suggest, then, that the murmur may be produced when the auricular contraction sends the blood through a mitral orifice of normal size into a dilated ventricle; that is to say, when there is stenosis of the mitral orifice relatively to the ventricle, though there may be no actual stenosis of the orifice. If this explanation be correct we have a method of distinguishing between the Austin Flint murmur and that due to mitral obstruction. The former will only appear when the ventricle begins to fail, and will disappear when compensation is restored; while the latter disappears when failure develops and reappears when the left auricle and right ventricle recover their power and function. I have been able, on more than one occasion, to watch this sequence of events.

### Murmurs that may be Confused with that of Aortic Incompetence.

1. *Pericardial Friction.*—Sometimes, though rarely, a single murmur, diastolic in time and produced by friction between the visceral and parietal pericardium, may simulate that due to incompetence of the aortic valves. This murmur, as a rule, seems very superficial, is modified markedly by pressure with the bell of the stethoscope, and generally does not begin exactly at the same time as the ventricular diastole. The absence of the concomitant signs of aortic incompetence usually serves to establish the diagnosis.

2. *Cardio-Pulmonary.*—Occasionally a murmur is produced by the mechanical displacement of air from the lung by the alternately contracting and dilating heart. It is possible for a murmur so produced, if diastolic in time, to simulate somewhat closely the murmur of aortic incompetence. Such a murmur is much modified by the respiratory movements, and may be heard only in one position of the thorax. Concomitant signs of aortic incompetence, of course, will be absent.

3. When the mitral valve is obstructed a murmur is sometimes heard which ceases at an appreciable interval before the first sound. Here again the murmur may at times be confused with the diastolic murmur of aortic regurgitation. This murmur is probably to be explained by the fact that owing to fibrosis of the bundle of His, there is delay in the passage of the contraction wave from the auricle to the ventricle. The auricle contracts well and produces a murmur, but the ventricular systole does not commence immediately the auricular systole terminates. It may be recognised by the presence of other signs of mitral obstruction and the absence of evidence of aortic regurgitation.

4. A short soft diastolic murmur may sometimes be heard in the pulmonary area following the second sound, when the mitral valve is obstructed but the aortic valve competent. Graham Steell has termed this the murmur of high pressure in the pulmonary artery. He suggests that it indicates incompetence of the pulmonary valve due to temporary dilatation of the pulmonary artery, which vessel is so often atheromatous when the mitral valve has been long stenosed. The murmur, as he points out, comes and goes according to the degree of blood-pressure within the pulmonary vessels. When present it is generally associated with a double second sound.

5. A murmur diastolic in time may occasionally result from permanent incompetence of the pulmonary artery. This is best heard in the pulmonary area and over the lower half of the sternum. Pulmonary incompetence is an extremely rare lesion, but may sometimes develop in association with congenital lesions of the pulmonary valve, with septic or malignant endocarditis, and with pressure on the pulmonary artery from without, as in aneurysm of the neighbouring sinus of Valsalva. Diagnosis of these conditions can only be established by very careful investigation.

6. Finally a diastolic murmur in very rare cases may occur in association with a thoracic aneurysm. The diagnosis of such a condition may be of extreme difficulty. Careful consideration of all the physical signs and examination with the *x*-rays may determine the truth.

I have now discussed with you the cause, character, and diagnosis of the murmur that may be found in association with incompetence of the aortic valve. It remains for me to urge the importance of close and careful consideration of all the signs before arriving at a diagnosis. Probably there is no cardiac lesion which is more commonly overlooked or incorrectly suspected by those who rely chiefly on the use of the stethoscope in the diagnosis of heart disease, than aortic regurgitation.

